# COVID-19 vaccine hesitancy among breast cancer patients in East Avenue Medical Center

**DOI:** 10.1192/j.eurpsy.2022.791

**Published:** 2022-09-01

**Authors:** O.K. Castro, S.F. Ty

**Affiliations:** East Avenue Medical Center, Internal Medicine, Quezon City, Philippines

**Keywords:** Covid-19, breast cancer, vaccine hesitancy

## Abstract

**Introduction:**

Vaccine hesitancy has been an ongoing complex public issue in the Philippines posing threats to progress against preventable outbreaks and significant morbidity and mortality from COVID-19. Patients with cancer were not included in the initial vaccine trials against COVID-19 hence the plausible explanation behind vaccine hesitancy in this population. This study attempts to determine the factors affecting a patient diagnosed with breast cancer to receive COVID-19 vaccine based on constructs from the Health Beliefs Model (HBM).

**Objectives:**

To determine the factors affecting a Filipino diagnosed with breast cancer to receive COVID-19 vaccine, namely; perceived susceptibility and severity to COVID-19 and perceived benefits and barriers to getting a vaccination against COVID-19.

**Methods:**

A single- center, descriptive, cross-sectional study in patients diagnosed with breast cancer was conducted to assess COVID-19 vaccine hesitancy.

**Results:**

A total of 85 eligible breast cancer patients were included in the analyses. Age, socio-economic factors, and presence of co-morbidities and metastasis were not significantly associated with COVID-19 vaccine hesitancy. Concerns on efficacy, safety, faulty or fake vaccine, as well as if the vaccine was taken by many in the public were significantly associated with hesitancy (p <0.05) when taken as individual factors. The perception of COVID-19 vaccine safety under the perceived barriers construct was found to be the only significant factor to predict vaccine hesitancy (OR= 4.737, CI 1.75, 12.82).

**Conclusions:**

Interventions that focus on perceived barriers are most crucial in order to increase vaccination rate among breast cancer patients.

**Disclosure:**

No significant relationships.

